# Emptying the fridge syndrome: a case of nocturnal food craving associated with peak perampanel concentration

**DOI:** 10.1016/j.ebr.2025.100821

**Published:** 2025-08-10

**Authors:** Paola Vassallo, Josemir W. Sander

**Affiliations:** a UCL Queen Square Institute of Neurology, London WC1N 3BG & Chalfont Centre for Epilepsy, Chalfont St Peter SL9 0RJ, UK; b Stichting Epilepsie Instellingen Nederland (SEIN), 2103 SW Heemstede, the Netherlands; c Department of Neurology, Leiden University Medical Centre, Leiden, the Netherlands; d Department of Neurology, West China Hospital, Sichuan University, Chengdu 610041 Sichuan, China

**Keywords:** Perampanel, Epilepsy, Therapeutic drug monitoring, Adverse effects, Food craving

## Abstract

Perampanel, a non-competitive AMPA receptor antagonist, is widely used in treating focal and generalised epilepsy. We report the case of a 26-year-old man with pharmaco-resistant focal epilepsy who developed nocturnal compulsive food-seeking behaviour after off-label titration of perampanel to 24 mg/day. Symptoms resolved entirely after dose reduction, supporting a possible causal relationship. Pharmacokinetic data showed a 26 % increase in serum levels between near-trough and peak, potentially explaining the timing of nocturnal symptoms. This case underscores the importance of recognising atypical behavioural adverse events. Direct questioning is essential, as individuals may not spontaneously report such symptoms due to stigma or fear of losing seizure control.

## Highlights

• Off-label antiseizure drug use is common but lacks full safety or efficacy data.
• High-dose perampanel may cause nocturnal compulsive food-seeking behaviour.
• Adverse events might go unreported without proactive questioning by clinicians.
• Effective but poorly tolerated drugs may warrant cautious rechallenge.

## 1 Introduction

The heterogeneity of epilepsy often requires the off-label use of antiseizure medications (ASMs), especially when people do not achieve seizure freedom. Off-label use refers to the administration of a drug outside its approved indications, dosage or age. Off-label use provides additional therapeutic options but also represents several challenges. Clinical data on safety and efficacy are often lacking, resulting in uncertainties regarding dosing and the potential risk of adverse events.

We describe here a peculiar dose-related adverse event in a young man taking a high dose of perampanel for drug-resistant epilepsy.

## 2 Case presentation

A right-handed 26-year-old man with a history of focal drug-resistant epilepsy presented to the clinic with what he described as a “raiding the fridge at midnight” compulsion. He was diagnosed with epilepsy at age 12 and experienced psychic or non-specific auras, occasionally progressing to focal seizures with impaired consciousness and generalised tonic-clonic seizures. Neurological examination was unremarkable. Brain MRI showed likely post-traumatic lesions in the right anterior temporal pole and basal frontal region, possibly secondary to a childhood pool diving accident. Ambulatory EEG showed interictal intermittent slowing in the right anterior quadrant and an ictal pattern of bursts of generalised spike-and-waves with right hemispheric predominance. Cognitive performance was within the normal range on the baseline neuropsychometric assessment.

Various antiseizure medications (ASMs), including sodium valproate, lamotrigine, zonisamide, topiramate, clobazam, and levetiracetam – prescribed by his paediatrician and local neurologist, either as monotherapy or in various combinations – resulted in unsatisfactory seizure control. Lamotrigine increased seizure frequency, and high doses of levetiracetam (4500 mg/day) caused irritability. In 2016, perampanel was added to levetiracetam 4000 mg/day and gradually titrated up to 24 mg in 2 mg increments to achieve seizure freedom. This resulted in a marked reduction in seizure frequency from monthly events to just one or two yearly. No drug monitoring was performed during the titration phase.

At a perampanel dose of 14–16 mg, taken once daily at night, he started having unusual nocturnal episodes about two hours after taking the evening dose. His mother observed behavioural changes at night, including slurred speech, a flustered demeanour, irritability, anger, and an overwhelming craving to eat, which he could not control. He had only partial recollection of these events as flashbacks; however, he was awake and able to interact properly when they occurred. While overall appetite increased, episodes of food craving occurred only at night, very frequently, but not every single night.

His compulsive behaviour was strictly food-related; he did not exhibit similar tendencies in gaming, shopping, drinking or sports. If access to the fridge was restricted, he would resort to ordering food or even attempting to leave the house, once climbing out a window in the middle of the night to buy food. These episodes led to considerable financial strain and weight gain, particularly after the perampanel dose was increased to 18 mg daily. Fearful of losing seizure control, he hesitated to disclose these symptoms to others, as perampanel had allowed him to lead a more normal life.

## 3 Case resolution

The individual was admitted to the hospital for observation during medication adjustment.

**Fig. 1:**
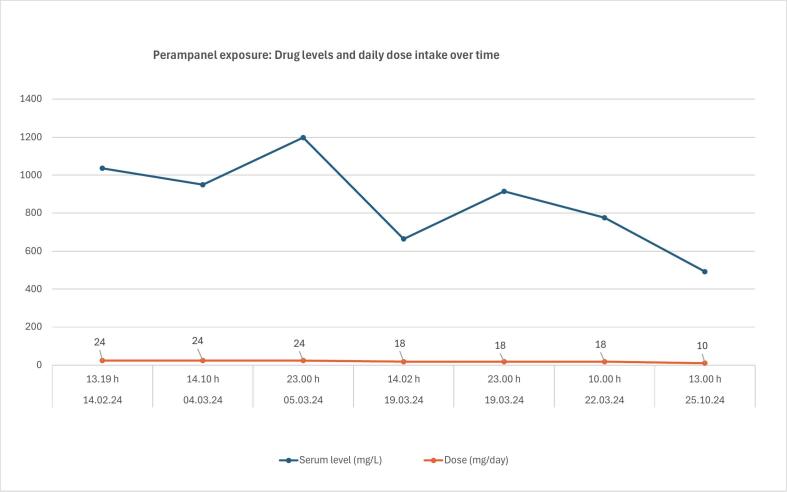
Perampanel exposure: serum levels (mg/L) on daily dose intake are dispayed with date and time collection.

Perampanel near-trough and peak serum levels were measured during admission and later during outpatient follow-up. During inpatient monitoring, serum levels increased by 26 % (from 950 ug/l to 1198 ug/l) between near-trough and peak levels. The latter was obtained 48 h after a dose reduction (from 24 mg to 22 mg) (see Table 1 and Fig. 1). For logistical reasons, actual trough levels – measured one hour before evening daily dose – could not be obtained. However, due to perampanel’s long half-life, near-trough levels are expected to closely approximate true trough levels.

**Table 1:** Perampanel, levetiracetam, and cenobamate daily dose (mg/day), time of intake, and serum levels, with date and collection time. Perampanel serum reference interval 199–1001 ug/L, levetiracetam serum reference interval 12–46 mg/L, cenobamate reference interval 5–35 mg/L according to NCT02535091.

Date and time of collection	Perampanel		Levetiracetam		Cenobamate	
	daily dose (mg)	Serum level (mg/l)	daily dose (mg)	Serum level (mg/l)	daily dose (mg)	Serum level (mg/l)
14.02.2024 at 13:19	24	1036	4000	30		
04.03.2024 at 14:10	24	950	4000	49		
05.03.2024 at 23:00	24	1198	4000			
19.03.2024 at 14:02	18	664	4000			
19.03.2024 at 23:00	18	915	4000			
22.03.2024 at 10:00	18	776	4000			
25.10.2024 at 13:00	10	492	4000	32	200	9.5

Compulsive eating was not observed during or after the inpatient stay but perampanel dose was decreased early and rapidly during this period.

Following a reduction in perampanel to 10 mg and the addition of cenobamate titrated to 200 mg, the nocturnal craving episodes resolved completely. Overall appetite normalised, and no further adverse events – whether food related or not– were reported. He lost considerable weight over the following months. His sleep quality improved, while seizure frequency returned to the pre-perampanel period – up to one generalised and several focal seizures per month – prompting presurgical assessment, which he is currently awaiting.

The partial recollection of the events, along with their full resolution after dose reduction, supports the hypothesis of a possible perampanel-induced adverse event rather than a primary sleep disorder. Additionally, normal psychometric tests argued against behavioural issues due to frontal lobe dysfunction. A rechallenge with perampanel was not considered, due to the significant burden on quality of life.

## 4 Discussion

Perampanel, a broad-spectrum ASM, acts as a selective non-competitive AMPA antagonist. As such, it may influence mesolimbic reward pathways regulating food-seeking behaviour and also hypothalamic appetite regulation circuits.

Permapanel is rapidly absorbed orally, exhibits linear pharmacokinetics, and reaches peak concentration between 15 min and 2 h, with a half-life of over six days. Metabolism primarily occurs in the liver via cytochrome P450 [1]. The usual prescribed dose for adults ranges from 2 to 12 mg/day, with a serum reference range of 0.25–2.85 μmol/L (0.1–1 mg/L). While generally well-tolerated when titrated slowly, perampanel may cause neurological, dose-dependent adverse events such as dizziness, fatigue, and sleepiness [2,3].

Behavioural and psychiatric adverse events have been reported, particularly in acute intoxication. These range from prolonged unresponsiveness [4] to aggressiveness with zombie-like behaviour [5], or at doses exceeding 8 mg/day, especially in individuals with pre-existing psychiatric conditions or those concurrently taking other ASMs like topiramate [6].

Increased appetite and weight gain, particularly in children on polytherapy [7], are well-documented, with one reported case of acute food aversion in a young woman after a dose increase [8]. Perampanel-induced weight gain has also been documented in a recent meta-analysis evaluating medication-related adverse events in individuals taking up to 12 mg/day. The mechanism of action for weight gain is not precise, whether it is secondary to increased food intake or an idiosyncratic effect [2]. However, administration of AMPA receptor antagonists in the nucleus accumbens has been shown to increase hedonic feeding and may contribute to overeating in animal models [9].

This novel observation of consistent nocturnal food-craving behaviour linked to high-dose perampanel expands the sparse evidence available on perampanel dose-related adverse events. There is a compulsive component that distinguishes this from general appetite changes, including goal-directed food-seeking behaviour (e.g., climbing out of windows). There is also a circadian pattern with the symptoms closely linked to pharmacokinetic peaks rather than constant hyperphagia. Symptoms emerged at doses well above the suggested maximum, resolving completely with dose reduction, aligning with the known dose-dependent neuropsychiatric effects of perampanel.

A pharmacokinetic explanation appears plausible, linking perampanel absorption and concentration to the timing of his food craving. Perampanel serum levels are dose-dependent [1]. The observed 26 % increase in serum concentration between the near-trough and peak levels, days after dose adjustment, suggests that actual peak levels during episodes were likely even higher than those measured. The timing of these peaks is also significant. Perampanel typically reaches peak serum levels 0.25 to 2 h post-dose. Given that he often took his medication after night shifts in a fasting state, rapid absorption would have been enhanced, leading to even higher concentrations. Serum levels were likely even higher during the food craving episodes, especially since he usually took the medication post-night shifts in a fasting state, potentially enhancing rapid absorption. This aligns with the onset of symptoms approximately two hours after his late evening dose, strongly suggesting that the food-craving episodes coincided with these elevated drug levels.

There are some limitations to our assumptions, including the absence of peak serum level data at the highest dose and the potential for confounding by concomitant levetiracetam at a high dose. We also didn’t rechallenge Perampanel at a later stage, nor did we attempt to split the dose to avoid a high peak concentration. Thus, this single case report requires validation through larger studies.

Direct questioning and semi-structured interviews during follow-up visits are essential, as individuals may be reluctant to report such symptoms openly. Given the limited tolerability and safety data beyond approved therapeutic doses, any off–label or supratherapeutic dosing should be approached with caution, and the threshold for adverse-event monitoring should be correspondingly lower.

Beyond its clinical implications, this case also raises ethical considerations regarding rechallenge with effective but poorly tolerated medications. In this case, the balance between efficacy and tolerability was unfavourable, yet perampanel might be reconsidered in the future if other treatment options – including epilepsy surgery – fail.

## 5 Consent for publication

Consent obtained.

## Funding sources

The Authors do not report funding related to this report.

## CRediT authorship contribution statement

**Paola Vassallo:** Writing – review & editing, Writing – original draft, Methodology, Formal analysis, Data curation, Conceptualization. **Josemir W. Sander:** Conceptualization, Data curation, Supervision, Writing – review & editing.

## Ethics approval

Ethical approval is not required for the publication of case reports.

## Declaration of competing interest

The authors declare that they have no known competing financial interests or personal relationships that could have appeared to influence the work reported in this paper.

## Data Availability

No data is available beyond what is reported.
